# Extract of *Aronia melanocarpa*-modified hemostasis: in vitro studies

**DOI:** 10.1007/s00394-014-0653-8

**Published:** 2014-01-23

**Authors:** Joanna Sikora, Magdalena Markowicz-Piasecka, Marlena Broncel, Elżbieta Mikiciuk-Olasik

**Affiliations:** 1 Department of Pharmaceutical Chemistry and Drug Analyses, Medical University of Lodz, Muszynskiego 1, 90-151 Lodz, Poland; 2 Department of Internal Diseases with Clinical Pharmacology, Medical University of Lodz, Kniaziewicza 1/5, 91-347 Lodz, Poland

**Keywords:** Adhesion, Aggregation, Fibrinolysis, Thrombin activity, Plasmin activity, *Aronia melanocarpa*, Chokeberry

## Abstract

**Purpose:**

*Aronia melanocarpa* has an extremely high content of procyanidins and anthocyanins. The multidirectional benefits of consumption of these berries are widely reported. Although numerous studies confirmed the influence of polyphenols on various stages of hemostasis, the exact mechanism of this phenomenon is not understood. The aim of our study was to evaluate the in vitro effect of *A. melanocarpa* extract on various parameters of hemostasis.

**Methods:**

Adenosine 5′-diphosphate (ADP)-induced aggregation was measured with turbidimetric method. Spontaneous and ADP-activated platelet adhesion were investigated using a colorimetric method. The global assay of coagulation and fibrinolysis was performed with the use of optical clotting and lysis (CL) test. Thrombin (0.5 IU/mL) and tissue plasminogen activator (60 ng/mL) were used to obtain a CL curve. The activity of thrombin and plasmin was determined by means of chromogenic substrate (S-2238, S-2251)

**Results:**

The aronia extract contributed to the reduction in spontaneous and ADP-activated platelet adhesion. A significant increase in overall potential of CL as well as significant changes in key parameters of these processes (*T*_t_—thrombin time, *F*_vo_—initial plasma clotting velocity, and *L*_max_—maximum lysis) was reported. Chokeberry extract significantly inhibited the amidolytic activity of thrombin and plasmin.

**Conclusion:**

Our in vitro findings indicate a complex mechanism of influence of chokeberry polyphenols on platelet activity and the overall potential of CL. We confirmed that chokeberry inhibits the amidolytic activity of thrombin. It was demonstrated for the first time that chokeberry polyphenols inhibit the amidolytic activity of another serine protease, i.e., plasmin, which is the main fibrinolytic enzyme. Furthermore, our research points out a significant contribution of other plasma components and fibrinogen in the modulation of hemostasis by polyphenols.

## Introduction

Many scientific studies support a correlation between the amount of plant-derived products and the lower risk of developing cardiovascular conditions and other diseases associated with oxidative stress. In the last decade, polyphenol plant compounds aroused particular interest of researchers mainly due to their powerful antioxidant properties as well as novel and still-being-discovered pleiotropic properties. It appears that among all antioxidants, polyphenols represent the strongest group. To date, scientists described more than eight thousands of natural polyphenols with very high diversity, ranging from simple structures, such as carbolic acid, to highly complex and polymerized molecules [1]. Berries, among them *Aronia melanocarpa*, are a very good source of polyphenols. Aronia, popularly known as the chokeberry, originates from North America and east Canada, but now it is also popular in Europe. Chokeberry fruits have extremely high content of procyanidins, anthocyanins, and phenolic acid, and because of its powerful antioxidant properties, Aronia is also the object of numerous scientific studies and publications [2].

Our previous studies conducted among patients with metabolic syndrome have shown the multidirectional benefits of supplementation of chokeberry extract. First of all, we reported a decrease in blood pressure and lipid levels, two very important risk factors for atherosclerosis supporting metabolic syndrome, along with a decrease in the concentration of endothelin-1. We also confirmed the antioxidant activity, the best described property of polyphenols. We found the increased activity of antioxidant enzymes such as CAT [EC 1.11.1.6], SOD [EC 1.15.1.1], and GSH-Px [EC 1.11.1.9] and reduced erythrocyte membrane lipid peroxidation after 2 months of chokeberry extract supplementation [3]. Our recent studies focused on the impact of chokeberry supplementation on platelet activity and on parameters of coagulation and fibrinolysis. Platelet hyperactivity and other disorders of hemostasis frequently occurring among patients with metabolic syndrome may result in embolic–thrombotic risk. Our studies have shown that 2 months of regular supplementation with polyphenols results in normalization of hemostatic parameters in patients with metabolic syndrome and favorably modifies platelet aggregation, albeit to a smaller extent [4]. Similar beneficial decrease in the platelet aggregation was observed in people in whom newly diagnosed hyperlipidemia was treated with statins, which are known for their numerous pleiotropic effects [5].

Although numerous in vitro studies confirmed the influence of polyphenols on various stages of hemostasis, the exact mechanism of this phenomenon is not understood [4, 6]. Many factors are involved in maintaining a balance between coagulation and fibrinolysis: vascular endothelium, blood cells (mainly platelets, but also the white blood cells), and coagulation factors—soluble plasma protein, in particular, serine proteases [7]. The encouraging results of previous clinical studies showing the beneficial effects of chokeberry extract prompted us to further research in order to establish whether the observed changes result from a direct impact on selected parameters of hemostasis or rather an indirect in vivo interaction of polyphenols with multiple components of hemostasis.

The aim of this study was to evaluate the in vitro effect of *A. melanocarpa* extract on parameters of hemostasis, such as platelet adhesion and aggregation, clot formation and fibrinolysis, thrombin and plasmin generation and activity.

## Materials and methods

### Reagents

Adenosine 5′-diphosphate (ADP), human fibrinogen, and urokinase-type plasminogen activator (u-PA) were obtained from Sigma-Aldrich (Munich, Germany), thrombin was purchased from Biomed (Lublin, Poland), and recombinant tissue plasminogen activator (t-PA) from Boehringer (Ingelheim, Germany). Chromogenic substrates—S-2238 (H–D-Phe-Pip-Arg-pNA) and S-2251 (H–D-Val-Leu-Lys-pNA)**—**were purchased from Chromogenix (Milan, Italy). Tris-buffered saline (TBS, pH 7.4), PBS, buffer A (pH 7.4), calcium chloride, and other chemicals were obtained from Polish Chemical Reagents (Gliwice, Poland). *Aronia melanocarpa* extract (AM) was purchased from Agropharm SA (Poland). This extract contained ca. 50 mg of total polyphenols, including a minimum of 15 mg of anthocyanins: 3-*O*-cyanidin-galactoside (64.5 %), 3-*O*-cyanidin-arabinoside (28.9 %), 3-*O*-cyanidin-xyloside (4.2 %), and 3-*O*-cyanidinglucoside (2.4 %). Aqueous solutions of extract were used within these studies. Solutions at appropriate concentrations were prepared immediately before the test.

### Material

#### Sample preparation

Blood was taken in the morning from fasting patients suffering from metabolic syndrome. All subjects gave their written informed consent prior to participating in the study. The study was approved by the Bioethical Committee of the Medical University of Lodz (No. 241/06/KB). The detailed inclusion and exclusion criteria were previously described (4). Blood was collected into vacuum tubes containing 3.2 % buffered sodium citrate. Platelet-rich plasma (PRP) for platelet analysis was obtained by blood centrifugation (150×*g*, 10 min, room temperature). Platelet-poor plasma (PPP) was obtained by subsequent centrifugation of PRP (2,500×*g*, 20 min, 4 °C). PPP was stored at minus 30 °C until further analysis. The platelet count in PRP was adjusted to 1.8 × 10^8^ platelets. To preserve the platelet discoid shape, PRP was restored in a water bath at 37 °C during a resting period of 30 min. Platelets for adhesion analysis were received by centrifugation of the PRP (700×*g*, 15 min, RT) and suspending the platelet sediment in buffer A (pH 7.4; 145 mmol/L NaCl; 5 mmol/L KCl; 10 mmol/L HEPES; 0.5 mmol/L Na_2_HPO_4_; 6 mmol/L glucose; 0.2 % bovine serum albumin). The platelet count in the PRP and platelet suspension was estimated routinely by means of photometry (*λ* = 800 nm) [8].

#### Platelet adhesion assay

Spontaneous and ADP-activated platelet adhesion to human fibrinogen (2 mg/mL) coated in multi-well microplates was investigated using a microplate reader (Elx 800; Bio-Tek Instruments Inc., Winosski, Vermont, USA) at 405 nm as described previously [9]. In the case of activated adhesion, 10 μL ADP (1 mmol/L) was added to stimulate platelets. The 45 μL of platelet suspension (2.5 × 10^6^ platelets/mL) was added into each well. The examined sample was pre-incubated at 37 °C for 3 min with aronia extract (range 0.5–100 μg/mL). Each sample had its own control (NaCl was added instead of the extract). After incubation, non-adhered platelets were carefully rinsed. The number of adherent platelets was estimated by measuring acid phosphatase activity—an unreleased platelet enzyme with stable activity independent of their stimulation. The 100 % of platelet adhesion was recognized as activity of acid phosphatase corresponding to 1.13 × 10^5^ platelets/well.

#### Platelet aggregation assay

Aggregation of platelets in PRP was measured with turbidimetric method using a Cecil CE2021 spectrophotometer (37 °C, with stirring; Cecil, London, UK). Aggregation curves were triggered by the addition of ADP (10 μmol/L) and were recorded and evaluated using our original computer software [10]. It enables us to estimate five parameters of platelet aggregation: maximal aggregation (*A*_max_), initial velocity (*v*_0_), the time needed to reach maximal aggregation (*T*_max_), the aggregation level after 5 min (*A*_5min_) from *A*_max_ (which enables to estimate disaggregation), and platelet shape change (PSC) (which is only a rough estimate of this process).

#### Test of clot formation and lysis (CL-test)

The previously described clotting and lysis (CL) test with our original computer software was used in present study [11]. The test was based on the assumption of the global assay of coagulation and fibrinolysis with the use of optical measurements of transmittance changes. To obtain a CL curve, thrombin (final concentration 0.5 IU/mL) and t-PA (final concentration 60 ng/mL) were added to PPP diluted three times in TBS-buffer. Curves were recorded continuously (37 °C, with stirring; Cecil CE 2021 spectrophotometer; Cecil, London, UK) and evaluated by the computer software. The following kinetic parameters of the examined processes were calculated: *C*_AUC_—overall potential of clotting, *T*_Gt_—thrombin generation time, and CL_AUC_—overall potential of clot formation and lysis; phase I (clot formation): *T*_t_—thrombin time, *F*_max_—maximum clotting, *T*_f_—plasma clotting time, *F*_vo_—initial plasma clotting velocity, and *S*_r_—area under the clot formation curve; phase II (forming a stable clot): *T*_c_—clot stabilization time and *S*_c_—area under the curve of a stable clot formation; phase III (fibrinolysis): *L*_max_—maximum lysis, *T*_l_—fibrinolysis time, *L*_vo_—initial clot fibrinolysis velocity, and *S*_f_—area under the fibrinolysis curve [11].

#### Thrombin generation and activity

The activity of thrombin was determined by measuring the hydrolysis of chromogenic substrate S-2238 (H–D-Phe-Pip-Arg-pNA). In the first stage, thrombin generation was carried out by incubation (20 min, 37 °C) of 150 μL of plasma, 150 μL of CaCl_2_ (25 mmol/L) and 150 μL of NaCl (154 mmol/L). After the removal of the clot, the supernatant was used to measure the activity of the generated thrombin. For this purpose, 20 μL of supernatant, 130 μL Tris-imidazole (0.025 mmol/L), 90–100 μL of NaCl (154 mmol/L), and 10 μL of chokeberry extract (0.5–100 mg/mL) were added to the wells of microtiter plates. After 3 min of incubation, 10 μL of the chromogenic substrate S-2238 (1 mmol/L) was added to the sample. The kinetic measurements were performed for 5 min at a 405 nm wavelength. Thrombin activity was calculated on the basis of a calibration curve of the thrombin activity ratio of 0.1–4 IU/mL.

#### Plasmin generation and activity

The generation and activity of plasmin were evaluated on the basis of the continuous measurement of plasmin-mediated proteolysis of chromogenic substrate (S-2251; H–D-Val-Leu-Lys-pNA) using a Cecil CE2021 spectrophotometer (Cecil, London, UK). For this purpose, 300 μL of plasma diluted three times with TBS (pH 7.4) was incubated (15 min, 37 °C) with 10 μL of chokeberry extract (2.5, 5, 10, 20, 100 μg/mL) and 10 μL of t-PA (35 ng/mL) or 10 μL u-PA (2 IU/mL). Then, 100 μL of S-2251 substrate (5 mmol/L) was added to the sample, and measurements (5 min) of the absorbance were started at a 405 nm wavelength. Registration and evaluation of the results were performed using the software for kinetic studies (DATA STREAM CE3000 5.0). The activity of the generated plasmin was expressed as the rate of the enzymatic reaction (A/min). Plasmin activity, determined by this method with the Standard Human Plasma for coagulation tests in normal range (Dade Behring, Marburg, Germany), amounted to 0.084 ± 0.003 A/min (the coefficient of variation = 4.2 %) or 0.147 ± 0.005 A/min (the coefficient of variation = 3.4 %), if induced by t-PA or u-PA, respectively.

### Data analysis

Percent of inhibition was calculated on the basis of the equation [(*A*_0_ − *A*_IC_) × 100 %]/*A*_0_, where *A*_0_—enzyme activity in the control sample, expressed as the rate of the enzymatic reaction (A/min) and *A*_IC_—enzyme activity in the sample with the extract of chokeberry expressed as the rate of the enzymatic reaction (A/min). The curve of percent inhibition of enzyme activity depending on extract concentration in the sample was prepared to determine the IC_50_ value. The IC_50_ value was calculated on the basis of the equation *y* = *a* × ln (*x*) + *b*.

All values were expressed as mean ± SD. Statistical tests were performed using a commercially available software package (Statistica 8.0). The Kolmogorov–Smirnov test was used to verify the normal distribution of continuous data. Depending on the distribution, the significance of intergroup differences was tested with the Wilcoxon signed-rank test or the paired *t* test. The *p* value <0.05 was considered statistically significant.

## Results

**Fig. 1:**
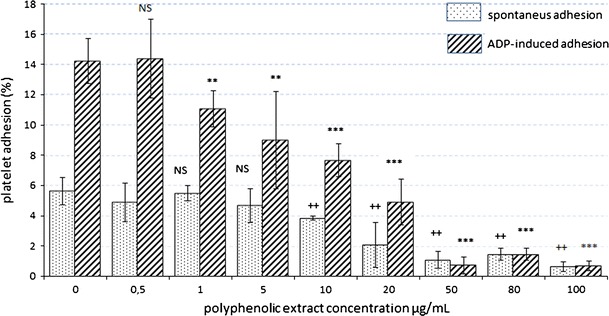
In vitro effect of aronia extract on spontaneous and ADP-induced platelet adhesion (%). **/++*p* < 0.05. ***/+++*p* < 0.001 versus control, *NS* not statistically significant

Incubation of platelet-rich plasma with chokeberry extract significantly influenced platelet reactivity. Even low concentrations of the extract (1 μg/mL) contributed to a 22 % reduction in the ADP-activated platelet adhesion to fibrinogen-coated surfaces (from 14.22 ± 1.5 to 11.08 ± 1.2 % of platelets introduced into the well). Medium to high concentrations in the range of 20–100 μg/mL caused more than 90 % inhibition of adhesion (less than 1.5 % of platelets introduced to the well). Moreover, the chokeberry extract reduced spontaneous adhesion to fibrinogen, but the effect was somewhat weaker. Only a 10 times higher concentration (10 μg/mL) produced a statistically significant decrease, and the highest concentrations resulted in approximately 80 % (about 1–2 % platelets introduced to the well) reduction in the capacity of spontaneous platelet adhesion (Fig. 1).

The effect of three concentrations (1, 10, 100 μg/mL) of chokeberry extract on ADP-induced platelet aggregation was also evaluated. Incubation with low concentrations of the extract (1 μg/mL) did not significantly affect kinetic parameters of the aggregation process, and a moderate effect was observed for platelet-rich plasma incubated with 10 μg/mL of chokeberry. The maximum aggregation (*A*_max_) and initial velocity (*v*_0_) of the process (Table 1) were reduced by approximately 30 %. The highest tested concentration (100 μg/mL) contributed to an almost complete inhibition of aggregation and significantly changed all the evaluated parameters, the magnitude of platelet shape change, and the time to reach *A*_max_. Longer incubation (15 min) of PRP with chokeberry extract (1–100 μg/mL) did not induce spontaneous aggregation.

**Table 1:** In vitro effect of aronia extract on kinetic parameters of ADP-induced platelet aggregation (mean ± SD)

ADP-induced platelet aggregation (PA) parameters	Control (*n* = 12)	Polyphenolic extract concentration (μg/mL)		
		1 (*n* = 10)	10 (*n* = 9)	100 (*n* = 7)
*A*_max_ (%T)	26.6 ± 12.9	28.6 ± 16.5	17.9 ± 11.1, *p* = 0.009	3.9 ± 3.3, *p* < 0.001
*v*_0_ (%T/min)	18.9 ± 13.0	16.9 ± 13.4	12.9 ± 13.0, *p* = 0.004	3.5 ± 3.0, *p* < 0.001
*T*_max_ (s)	356.5 ± 173.6	397.2 ± 165.4	395.6 ± 173.6	401.1 ± 156.6, *p* = 0.042
PSC_max_ (%T)	2.5 ± 3.1	3.5 ± 2.7	4.5 ± 3.1	4.9 ± 3.8, *p* = 0.003

*A*_max_ (%T) maximal aggregation, *v*_0_ (%T/min) initial velocity, *T*_max_ (s) time needed to reach *A*_max_, and PSC_max_ (%T) maximal platelet shape change

**Fig. 2:**
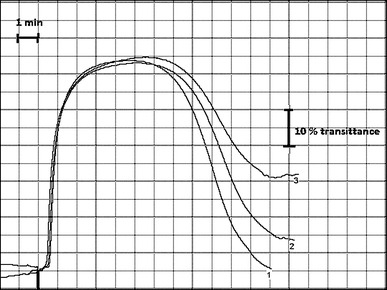
Effect of aronia on curve of clot formation and lysis: *1* control; *2* 33 μg/mL; and *3* 66 μg/mL of *Aronia melanocarpa* extract. *Bold line* on the graph represents, respectively, 1 min and 10 % of transmittance

The effect of chokeberry extract on the overall potential of the process of clot formation and fibrinolysis (CL) and the various kinetic parameters of the process is documented in Table 2. For both examined concentrations (33 and 66 μg/mL), a statistically significant increase in overall potential of CL, as well as significant changes in key parameters of these processes, such as *T*_t_—thrombin time, *F*_vo_—initial plasma clotting velocity, and *L*_max_—maximum lysis, was observed (Fig. 2). Incubation of plasma with high concentrations of the extract (100 μg/mL) caused a decrease in transmittance (up to 25 %) in some samples, disabling further investigations. The effect of chokeberry on the coagulation process under the influence of endogenous thrombin is summarized in Table 3. A statistically significant prolongation of the *T*_Gt_—thrombin generation time was documented in response to incubation with 66 mg/mL of the extract.

**Table 2:** Effect of aronia on kinetic parameters of overall potential of clot formation and lysis induced by high exogenous thrombin concentration and t-PA (mean ± SD)

Phase	Parameters	Control (*n* = 11)	Polyphenolic extract concentration (μg/mL)	
			33 (*n* = 11)	66 (*n* = 11)
I	*T*_t_ (s)	36.11 ± 3.88	33.84 ± 3.67, *p* = 0.005	30.01 ± 2.31, *p* = 0.0038
	*F*_max_ (%T)	49.25 ± 13.08	49.34 ± 10.89	50.84 ± 7.09
	*T*_f_ (s)	95.08 ± 29.82	94.35 ± 16.33	110.55 ± 23.78
	*F*_vo_ (%T/min)	105.31 ± 43.59	116.25 ± 44.62, *p* = 0.011	120.22 ± 29.88, *p* = 0.05
II	*T*_c_ (s)	401.92 ± 149.02	395.23 ± 70.99	408.00 ± 79.03
	*S*_c_ (%T × min)	329.59 ± 198.40	313.99 ± 82.29	332.68 ± 54.11
III	*L*_max_ (%T)	46.26 ± 12.14	42.92 ± 9.85	34.52 ± 7.0, *p* = 0.01444
	*T*_l_ (s)	257.15 ± 77.90	274.15 ± 50.75	281.64 ± 49.30
	*L*_vo_ (%T/min)	14.91 ± 5.12	13.42 ± 5.05	12.38 ± 6.86
	CL_AUC_ (%T × min)	507.04 ± 248.49	545.96 ± 122.23, *p* = 0.03	672.50 ± 94.75, *p* = 0.03

Phase I (clot formation): *T*_t_ thrombin time (s), *F*_max_ maximum clotting (%T), *T*_f_ plasma clotting time (s), and *F*_vo_ initial plasma clotting velocity (%T/min). Phase II (forming a stable): *T*_c_ clot stabilization time (s) and *S*_c_ area under the curve of a stable clot formation (%T × min). Phase III (fibrinolysis): *L*_max_ maximum lysis (%T), *T*_l_ fibrinolysis time (s), *L*_vo_ initial clot fibrinolysis velocity (%T/min), CL_AUC_ overall potential of clot formation, and lysis (%T × min); *p* = values versus control

**Table 3:** Effect of aronia on kinetic parameters of overall potential of plasma clotting induced by endogenous thrombin (mean ± SD)

Parameters	Control (*n* = 12)	Polyphenolic extract concentration μg/mL	
		33 (*n* = 11)	66 (*n* = 7)
*T*_Gt_ (s)	147.0 ± 47.7	150.5 ± 49.2	154.3 ± 45.9, *p* = 0.05
*F*_max_ (%T)	66.2 ± 14.5	63.5 ± 14.0	72.9 ± 10.3
*T*_f_ (s)	75.9 ± 26.4	99.0 ± 67.9	86.6 ± 29.1
*F*_vo_ (%T/min)	124.6 ± 60.6	132.9 ± 75.2	122.4 ± 51.7
*C*_AUC_ (%T × min)	1,108.5 ± 290.4	1,048.4 ± 254.2	1,206.1 ± 189.5

*T*_Gt_ thrombin generation time (s), *F*_max_ maximum clotting (%T), *T*_f_ plasma clotting time (s), *F*_vo_ initial plasma clotting velocity (%T/min), and *C*_AUC_ overall potential of clotting (%T × min); *p* = values versus control

**Fig. 3:**
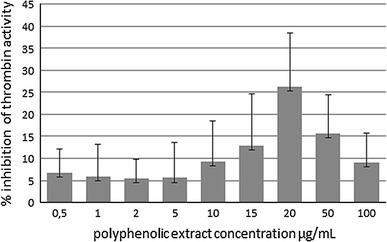
Influence of polyphenolic extract of aronia on % inhibition of amidolytic thrombin activity (data presented as mean with standard deviation; *n* = 8)

The effects of ten different concentrations of chokeberry extract (between 0.5 and 100 μg/mL) on thrombin amidolytic activity were examined. While an insignificant effect was observed in the case of low concentrations (0.5–5 μg/mL), a statistically significant inhibition of thrombin was documented for 10 μg/mL of the extract (Table 4). The most pronounced inhibition, corresponding to about 26 %, was observed in the case of incubation with 20 μg/mL of the extract (Fig. 3).

**Table 4:** Influence of aronia on amidolytic thrombin activity (IU/mL) (data presented as mean with standard deviation); *NS* not statistically significant, *p* = values versus control

	Control (*n* = 8)	Polyphenolic extract concentration (μg/mL) (*n* = 8)								
		0.5	1	2	5	10	15	20	50	100
Thrombin activity (IU/mL)	0.68 ± 0.05	0.63 ± 0.07, NS	0.66 ± 0.09, NS	0.65 ± 0.03, NS	0.66 ± 0.09, NS	0.61 ± 0.07, *p* = 0.004	0.60 ± 0.09, *p* < 0.001	0.52 ± 0.11, *p* < 0.001	0.56 ± 0.06, *p* < 0.001	0.61 ± 0.05, *p* = 0.002

**Fig. 4:**
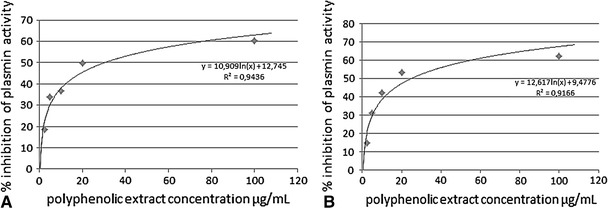
Influence of polyphenolic extract of aronia on % inhibition of plasmin activity: **a** t-PA induced. **b** u-PA induced (data presented as mean; *n* = 8)

We also examined the effect of aronia extract on plasmin activity induced by two endogenous plasminogen activators: t-PA and u-PA. The activity of plasmin, expressed as the velocity of the enzymatic reaction, is presented in Table 5. The t-PA-induced plasmin activity was significantly (*p* = 0.01) lower than that induced by u-PA. Similar results were obtained in the case of standard control plasma, in which the activity of plasmin equaled to 0.084 ± 0.003 A/min for t-PA and 0.149 ± 0.005 A/min (*p* = 0.006) for t-PA and u-PA, respectively. All studied concentrations of chokeberry extract (2.5–100 μg/mL) significantly inhibited the amidolytic activity of plasmin. We identified the concentrations of the extract that decreased the activity of this enzyme by 50 % (IC_50_): 30.4 and 24.8 μg/mL for t-PA- and u-PA-activated samples, respectively (Fig. 4a, b).

**Table 5:** Influence of aronia on amidolytic plasmin activity expressed as the rate of enzymatic reaction (A/min)

	Control	Polyphenolic extract concentration (μg/mL)				
		2.5	5	10	20	100
t-PA-activated plasminogen (A/min) (*n* = 7)	0.084 ± 0.040	0.072 ± 0.048, *p* = 0.001	0.061 ± 0.043, *p* < 0.001	0.056 ± 0.041, *p* = 0.001	0.044 ± 0.041, *p* = 0.001	0.034 ± 0.040, *p* = 0.002
u-PA-activated plasminogen (A/min) (*n* = 8)	0.148 ± 0.032	0.125 ± 0.028, *p* = 0.009	0.101 ± 0.033, *p* = 0.008	0.081 ± 0.034, *p* = 0.009	0.066 ± 0.034, *p* = 0.003	0.042 ± 0.011, *p* = 0.002

Data presented as mean with standard deviation; *p* = values versus control

## Discussion

Metabolic syndrome (MS) is characterized by central obesity, impaired glucose tolerance, dyslipidemia, and hypertension. Furthermore, MS was revealed to be associated with non-traditional cardiovascular disease risk factors, such as inflammatory processes, disorders of coagulation and fibrinolysis, and platelet hyperactivity [12].

The use of natural, commonly accepted, and widely available supplements rich in plant polyphenols represents one of such strategies. *Aronia melanocarpa* berries are one example of a natural source of pharmacologically essential substances [2, 13]. Polyphenols, predominantly flavonoids, such as procyanidins and anthocyanins, constitute the most important and most extensively studied group of active compounds contained in those fruits. Procyanidins are in general oligomeric and polymeric (epi)catechins formed from the association of several monomeric units: When 2–10 units are present, (epi)catechin oligomers are addressed, with over 10 units we speak of (epi)catechin polymers. Aronia contains exclusively homogeneous B-type procyanidins (whereby C4′C8 and/or C4′C6 bonds are the predominant types) with (−)-epicatechin as the main subunit monomer. The portion of catechin units is about 1.5 %. The procyanidins composition in Aronia is reported as the following: monomers (0.78 %), dimers (1.88 %), trimers (1.55 %), 4–6-mers (6.07 %), 7–10-mers (7.96 %), and >10-mers (81.72 %). *Aronia melanocarpa* berries are known as one of the richest plant sources of anthocyanins, mainly containing 4 cyanidin glycosides. In chokeberry fruits, the anthocyanins represent about 25 % of the total polyphenols. This class of flavonoids is responsible for the pigments that give berries their dark red, blue, and purple colors [2]. The polyphenolic compounds are responsible for the strong antioxidant properties of chokeberry. The total content of polyphenols depends on growth conditions and time of fruit harvesting and ranges from 2,000 to 8,000 mg for 100 g of dry weight. The polyphenols present in chokeberry fruits also include chlorogenic and neochlorogenic acids and small amounts of tannins [2].

Until recently, it was believed that polyphenolic compounds are not absorbed from the gastrointestinal tract. Currently, there are many studies [14] in which the concentrations of both anthocyanins and their metabolites in blood and urine were defined. Most of these studies are performed after single oral administration of small doses of anthocyanins in the form of juice or extract from chokeberry, and the maximum concentrations of anthocyanins detected in plasma were in micromolar (29–350 nmol/L) units. In the case of polymeric procyjaninidins which are hardly absorbed in the intestines, it is thought that their health-promoting actions result from the direct effect on the intestinal mucosa and protect it against oxidative stress or actions of carcinogens [13].

Moreover, aronia constitutes a source of sugar (10–18 %), pectins (0.6–0.7 %), sugar alcohol (sorbitol, parasorboside), and a small amount of fat (0.14 % of the fresh weight; mainly linoleic acid, glycerides, and phosphatidylinositol). Chokeberries are also a source of minerals (mainly K and Zn, small amounts of Na, Ca, Mg, Fe) and vitamins (vitamin B complex, vitamin C, niacin, pantothenic acid, folic acid, α- and β–tocopherol, and carotenoids) [2].

Blood platelets play a vital role during the complex process of physiological hemostasis. However, their hyperactivity, often observed in patients with cardiovascular diseases, is an essential component of the pathogenesis of atherosclerotic plaque. This is related to the increased tendency for platelet adhesion, aggregation, and degranulation of an array of active agents, which may also enhance endothelial dysfunction. Epidemiological studies have shown that a decrease in platelet reactivity can be achieved with proper diet rich in polyphenols. We have observed this beneficial effect of polyphenols on platelet reactivity in response to 4-week supplementation with polyphenol-rich extract from chokeberry in patients with metabolic syndrome [4].

In the present in vitro study, we evaluated the direct effect of chokeberry extract on the three key elements of platelet reactivity: spontaneous and ADP-activated adhesion and ADP-induced aggregation.

Adhesion constitutes the first stage of platelet activation, which determines the further course of hemostasis. Genetic and acquired adhesion defects may cause serious coagulation disorders. On the other hand, undesirable increase in adhesion is an important risk factor for atherosclerosis [15]. In contrast to platelet aggregation in solution, the platelet adhesion was suggested to not require pre-activation, and resting platelets appear to adhere to fibrinogen-coated surfaces. In the case of decreased perfusion, activated platelets adhere to the surface-immobilized fibrinogen faster and more extensively than resting platelets, a property which may be important in the pathogenesis of atherosclerotic plaque [16].

The spontaneous adhesion of platelets to fibrinogen is postulated as an indirect indicator of their condition in vivo.

Our study revealed that an average of 5.6 ± 0.9 % of total 1.13 × 10^5^ platelets from patients with metabolic syndrome showed spontaneous adhesion, if introduced into wells coated with fibrinogen (Fig. 1). After incubation with different concentrations of polyphenolic extract, the amount of spontaneously adhesive platelets decreased significantly, to 3.9 ± 0.4 % at a concentration of 10 μg/mL and to less than 2 % at concentrations higher than 20 μg/mL. In ADP-stimulated control samples, the adhesion was documented in the case of approximately 14.2 ± 1.5 % of platelets. After incubation with different concentrations of chokeberry extract, we reported a statistically significant concentration-dependent inhibition of ADP-induced adhesion. Compared to the results yielded by spontaneous adhesion, the effect has been observed at 10 times lower concentration (1 μg/mL; Fig. 1).

The effect of polyphenolic extracts of black chokeberry and grape seeds on spontaneous and thrombin-activated adhesion to fibrinogen and collagen was examined by Malinowska et al. [17]. Contrary to our study, authors [17] analyzed the platelets isolated from healthy volunteers and generated oxidative stress only once the samples were in in vitro environment, by incubating the platelets with homocysteine (HCY) or its more reactive form—homocysteine thiolactone (HTL). Malinowska et al. [17] showed that in vitro incubation of platelets from healthy donors with HCY and HTL, i.e., platelets submitted to generated oxidative stress, increases both the spontaneous and the activated adhesion. Chokeberry extract inhibited the thrombin-activated adhesion both in cases of the platelets exposed to oxidative stress and in cases of the platelets not incubated with HCV/HTL.

Olas et al. [6] compared different formulations of polyphenols using platelets from healthy volunteers. These authors also observed that chokeberry extract (5–50 μg/mL) exerted strong inhibitory effect on the adhesion of thrombin-activated platelets to collagen. In this case, the polyphenol extracts’ ability to inhibit the strongly activated platelets has to be emphasized, seeing collagen as a potent platelet agonist.

The results of both in vitro [6, 17] and in vivo studies [18, 19] suggest that chokeberry extract inhibits the aggregation induced by collagen or thrombin. This positive phenomenon is observed in both healthy volunteers and in patients who run the risk of developing either atherosclerosis [18, 19] or thromboembolic disorders associated with breast cancer [20].

Our in vitro study has shown that the chokeberry extract significantly inhibits ADP-induced platelet aggregation. This suggests that polyphenol preparations can directly block the ADP-dependent pathway of platelet activation. We revealed that extremely high concentrations of the extract (100 μg/mL) caused an almost complete inhibition of the aggregation. Indeed, two key kinetic parameters of the process were reduced: The maximum aggregation (*A*_max_) was 3.9 ± 3.3 % T versus 26.6 ± 12.9 % T in the control (*p* < 0.001) and the initial velocity *v*_0_ (% T/min) 3.0 ± 3.5 versus 18.9 ± 13.0 (*p* < 0.001), respectively. The incubation with 10 μg/mL of the chokeberry extract was reflected by slightly weaker, albeit still significant, inhibition of ADP-induced aggregation (*A*_max_ = 17.9 ± 11.1 (*p* = 0.009), *v*_0_ = 12.9 ± 13.0 (*p* = 0.004)), while the lowest concentration did not exert a significant effect on the analyzed test (Table 1).

The in vitro effect of polyphenols on platelet activity was to a certain extent confirmed in clinical trials. Our previous ex vivo study [4] also revealed decreased aggregation of platelets in response to 4-week treatment with Aronox. Erlund et al. [21] have shown that regular consumption of berries, including the chokeberry, significantly reduces platelet function measured with the use of analyzer evaluating occlusion of ADP-activated platelets to collagen. The study has proved that a diet rich in ingredients derived from chokeberry prolongs occlusion, which, indirectly, may be considered as reducing the platelets’ capacity of adhesion and aggregation [21].

During the next stage of our study, we analyzed the effect of polyphenolic extract on the overall potential of clot formation triggered by large doses of exogenous thrombin and fibrinolysis of the clot. Moreover, we assessed the endogenous thrombin generation process and the amidolytic activity of thrombin and plasmin.

We have noted a significant shortening of *T*_t_, i.e., the time elapsed from the addition of thrombin to initial formation of fibrin fibers, along with a significant increase in the initial velocity of clot formation (*F*_vo_) (Table 2). On the other hand, chokeberry extract slowed down the process of endogenous thrombin generation; interestingly, despite significantly extended *T*_Gt_, the initial velocity of clot formation (*F*_vo_) and other kinetic parameters of the overall clotting potential remained unchanged (Table 3). Additionally, we tested the impact of nine different concentrations of the chokeberry extract (0.5–100 μg/mL) on the amidolytic activity of endogenously generated thrombin. Although we observed a significant inhibition of the activity of thrombin, this process was not concentration dependent, and the degree of inhibition was relatively low (5–25 %; Table 4; Fig. 3).

Bijak et al. [22] revealed that the polyphenol preparations (chokeberry and grape seeds) show antithrombin and anticoagulant activity. The authors observed a significant decrease in the velocity of fibrin clot formation, resulting from incubation of thrombin with polyphenols, and an almost complete inhibition of thrombin amidolytic activity at as low concentration of the chokeberry extract as 50 μg/mL. However, this study was conducted on isolated fibrinogen and chokeberry extract was incubated with pure thrombin. In contrast, our study included citrate plasma and, in every experiment and prior to the analysis, different levels of chokeberry extract which were incubated in the plasma. Our results illustrate that the extract has a more complex effect on the process of thrombin generation and its activity than was previously considered. Furthermore, the results may be interpreted as an indication of direct chemical reactions between the ingredients of preparation and fibrinogen, as well as of a protective effect of other plasma proteins.

None of previously published research analyzed the effect of polyphenols on fibrinolysis. Our findings suggest that this process can be modulated by the incubation of plasma with aronia extract. Figure 2 shows the overall potential for clot formation and fibrinolysis before and after incubation with the extract. While the evident reduction in the maximum lysis (*L*_max_) can be observed in response to stimulation with the extract, the initial clot fibrinolysis velocity (*L*_vo_) remains unchanged. The observed effect may result from both the quantitative changes (reducing the activity of proteolytic enzymes) and the qualitative alterations of fibrin degradation products. We demonstrated a concentration-dependent effect of polyphenols on the amidolytic activity of plasmin, a key enzyme responsible for the lysis of fibrin. The activity of plasmin induced by two plasminogen activators, t-PA and u-PA, was assessed in plasma (Table 5). We observed a difference in the activity of generated plasmin already in the control samples. These differences stem from the mechanisms of activating plasminogen by these compounds. While u-Pa is a direct activator, t-PA is subject to extensive regulation, e.g., by plasminogen activator inhibitor (PAI). These findings may be the indicators of why a slight difference was obtained in the IC_50_ values (30.4 and 24.8 μg/mL for t-PA- and u-PA-activated samples, respectively; Fig. 4a, b). In the light of the results concerning the inhibition of plasmin activity, we may suspect that a protective action of extract may be the effect of chokeberry fruit ingredients on reduction in microbleeding in the gastrointestinal tract. However, further studies are needed, especially in vivo studies, to confirm this statement.

In our studies, we used a range of concentrations of the extract from chokeberry (0.5–100 μg/mL), consistent with the literature [14], and used by other authors in vitro [6, 16, 19, 21]. We relied also on the concentration of anthocyanins in the blood after consumption of different formulations of chokeberry and with regard to the percentage of anthocyanins content in the extract. Therefore, it seems that concentrations of 0.5–20 μg/mL in in vitro studies can correspond to those achieved in vivo. Concentration of 100 μg/mL appears to be extremely high, but there is insufficient data on concentrations that are obtained after long-term supplementation and high doses of chokeberry.

We are well aware of the potential limitations of our study. Firstly, due to the limited amount of biological material, we did not manage to evaluate the effect of a full range of panel concentrations of 0.5–100 μg/mL in studies using the traditional method. The presented concentrations were chosen during preliminary experiments as the most effective. Secondly, chokeberry extract itself, due to the high content of vegetable dyes, may affect the spectrophotometric measurements. We tried to eliminate this effect by introducing appropriate calibration procedures.

In conclusion, the results of our in vitro study suggest that the mechanism by which chokeberry polyphenols modulate platelet activity and the overall potential of CL is complex. Chokeberry extract directly inhibits both ADP-activation-dependent aggregation and adhesion and spontaneous adhesion. It cannot be excluded, however, that the beneficial effect of polyphenols observed in vivo is also associated with modulation of nitric oxide metabolism and antioxidant action [20]. Certainly, the extract’s effect on plasma coagulation and lysis is determined by an array of factors and their interactions. We confirmed that chokeberry inhibits amidolytic activity of thrombin. Moreover, we demonstrated for the first time that chokeberry polyphenols inhibit the amidolytic activity of plasmin, another serine protease being the principal fibrinolytic enzyme. The inhibitory effect of the chokeberry extract on the activity of plasmin, much stronger than the activity of thrombin, may also have benefits associated with reducing fibrinolysis both in circulatory system and in the gastrointestinal tract. Our research also points out the significant contribution of other plasma components and fibrinogen in the modulation of hemostasis by polyphenols. Understanding the exact influence of polyphenols on blood clotting and fibrinolysis still requires further study. Our opinion, based on the inhibitory effects of chokeberry polyphenols on the activity of two serine proteases, key hemostatic enzymes, confirmed by this study, is that it would be beneficial to examine the polyphenols’ effect on other coagulation factors, which also belong to the group of proteases.
